# What Is Health Information Quality? Ethical Dimension and Perception by Users

**DOI:** 10.3389/fmed.2018.00260

**Published:** 2018-09-20

**Authors:** Majed Al-Jefri, Roger Evans, Gulden Uchyigit, Pietro Ghezzi

**Affiliations:** ^1^School of Computing, Engineering and Mathematics, University of Brighton, Brighton, United Kingdom; ^2^Brighton and Sussex Medical School, Falmer, United Kingdom

**Keywords:** internet, information quality, ethics, online information, public health

## Abstract

**Introduction:** The popularity of seeking health information online makes information quality (IQ) a public health issue. The present study aims at building a theoretical framework of health information quality (HIQ) that can be applied to websites and defines which IQ criteria are important for a website to be trustworthy and meet users' expectations.

**Methods:** We have identified a list of HIQ criteria from existing tools and assessment criteria and elaborated them into a questionnaire that was promoted via social media and mainly the University. Responses (329) were used to rank the different criteria for their importance in trusting a website and to identify patterns of criteria using hierarchical cluster analysis.

**Results:** HIQ criteria were organized in five dimensions based on previous theoretical frameworks as well as on how they cluster together in the questionnaire response. We could identify a top-ranking dimension (scientific completeness) that describes what the user is expecting to know from the websites (in particular: description of symptoms, treatments, side effects). Cluster analysis also identified a number of criteria borrowed from existing tools for assessing HIQ that could be subsumed to a broad “ethical” dimension (such as conflict of interests, privacy, advertising policies) that were, in general, ranked of low importance by the participants. Subgroup analysis revealed significant differences in the importance assigned to the various criteria based on gender, language and whether or not of biomedical educational background.

**Conclusions:** We identified criteria of HIQ and organized them in dimensions. We observed that ethical criteria, while regarded highly in the academic and medical environment, are not considered highly by the public.

## Introduction

With the diffusion of the Internet, many have been concerned that, due to its unregulated and unfiltered nature, it could misinform or disinform the public. The lack of widely used search engines (Google was founded in 1998) left entirely up to the users which websites to trust among the relatively few ones (compared to the www in 2018) available. These concerns led to the development, in the late 1990s, of instruments and organizations to assess health information quality (HIQ) of websites, including the JAMA criteria (1), DISCERN (2), and the criteria for meeting the health-on-the-net (HON) code of conduct (3). These instruments were developed for different purposes: the JAMA and DISCERN tools were aimed at providing customers with instruments to assess websites (1, 2); the HON criteria are used by the HON foundation to certify health websites with the display of the HONCode quality seal, and this was originally aimed at organizations to help them develop websites (3). The criteria of HIQ considered by these three approaches are listed in Table 1.

**Table 1:** Established HIQ instruments and criteria.

**JAMA**	**HON**	**DISCERN**
Authorship Source attribution Ownership disclosure Currency	Authorship Attribution Privacy Complementarity Transparency Justifiability Financial disclosure Advertising policy	**Is the publication reliable?** 1. Are the aims clear? 2. Does it achieve its aims? 3. Is it relevant? 4. Is it clear what sources of information were used to compile the publication (other than the author or producer)? 5. Is it clear when the information used or reported in the publication was produced? 6. Is it balanced and unbiased? 7. Does it provide details of additional sources of support and information? 8. Does it refer to areas of uncertainty? **How good is the quality of information on treatment choices?** 9. Does it describe how each treatment works? 10. Does it describe the benefits of each treatment? 11. Does it describe the risks of each treatment? 12. Does it describe what would happen if no treatment is used? 13. Does it describe how the treatment choices affect overall quality of life? 14. Is it clear that there may be more than one possible treatment choice? 15. Does it provide support for shared decision-making?

*From (1–3)*.

There are no data available to know how many information seekers have used these tools to make assessments. On the other hand, the high number of citations in the scientific literature for the JAMA (1100) and DISCERN (600) tools indicate that these are also widely used, particularly the JAMA criteria, in academic research analyzing HIQ. It should be noted, however, that DISCERN was developed by an expert panel but then it was actually tested on 13 self-help group members (2).

An important issue, and one that is not assessed by the existing HIQ instruments, is whether websites informing the public on therapies mention therapies approved by regulatory agencies or public health authorities, or non-approved ones. Drug approval requires a high level of evidence of efficacy and benefit/risk ratio, an approach termed “evidence-based medicine” (EBM) (4). In a way, this is related to the reliability of the information. For instance, a website describing AIDS as a disease due to the HIV virus that can be treated with antiretroviral therapy is higher quality than one stating that AIDS is not due to a virus and should be treated with nutritional supplements (5).

Health information quality should be seen in the wider context of information quality (IQ) generally. The latter has been extensively studied for its applications in business and manufacturing. Information quality is generally considered as a concept with multiple dimensions (6); depending on an author's philosophical view-point information quality can have different attributes and characteristics (7, 8). Several studies have developed IQ frameworks based on the definition of IQ dimensions (6). The best known of these frameworks was developed by Wang (9) and Wang (10), based on a survey among 355 Masters in Business and Administration alumni, aiming to capture aspects of IQ that are important for consumers in the business field. A second study by the same group involved 52 information professionals from the financial, healthcare, and manufacturing sectors (11). These studies defined fifteen IQ criteria, that were grouped into four dimensions (9, 10) as shown in Table 2.

**Table 2:** Dimensions of IQ.

**Dimension**	**Criteria associated**
Intrinsic IQ	Accuracy, objectivity, believability, reputation
Accessibility IQ	Accessibility, security
Contextual IQ	Relevancy, value-added, timeliness, completeness, amount of information
Representational IQ	Interpretability, ease of understanding, concise representation, consistent representation

*Modified from (9, 10)*.

It is probably difficult to fit the HIQ criteria from Table 1, that are centered on trustworthiness and scientific correctness, into the theoretical framework of IQ dimensions in Table 2, that are borrowed from other fields. Recent studies have proposed a categorization of HIQ criteria into classical IQ dimensions focusing on IQ criteria identified through focus group, and focusing on the scientific content of webpages (12).

We undertook this project to define the IQ criteria and dimensions relevant to HIQ. To do so, we have used a mixed approach, identifying relevant HIQ criteria using a theoretical approach broadly based on the existing criteria, the JAMA score, HONcode and DISCERN, and an empirical approach, based on a questionnaire, to rank the importance of the various criteria to the end user. In particular, our aim was to evaluate user perceptions of HIQ criteria and their relative importance in trusting health-related websites. Criteria of HIQ were then classified in dimensions based on the existing literature and, using cluster analysis, the ranking by users.

## Methods

To design a questionnaire, we first identified relevant IQ criteria. These were based on the existing literature on HIQ, the instruments described above (Table 1) the standard IQ criteria listed in Table 2 and other studies (10, 13, 14). General criteria, such as correct spelling and grammar or the importance of the presence of multimedia or the ranking by the search engine were also included. Other questions are related to the content of the webpage, such as whether the webpage explains disease symptoms, therapies, how to take medications and their side effects, and if responders are wary of webpages offering quick solutions and miracle cures (we defined this as “hyperbole”). The respondents were also asked to rate importance that the information describes treatments based on evidence-based medicine or complementary medicine, as this question would be defining a criterion of reliability (from the scientific point of view) of the information.

The full list of HIQ criteria considered is provided in Table 3, that also reports the questions aiming at identifying the importance of those criteria in trusting a health-related website that were used in the questionnaire. The table also shows which criteria were derived from the ones in the known HIQ tools (JAMA, HON, DISCERN). For most of the criteria, the questions were formulated in the form “I trust a health webpage more if…” or “I prefer webpages that…” that were assessed using a 5-point Likert scale (5 = strongly agree, 4 = somewhat agree, 3 = neither agree not disagree, 2 = somewhat disagree, 1 = strongly disagree). Other questions were aiming at defining the demographics of the sample (gender, age, country, education, whether studying in a medically-related subject of not and others) or Internet usage (time spent, main search engine used, device used, how often they searched health information, whether searching symptoms or therapies). The entire questionnaire (42 questions) is available as Supplementary Online Information (Supplementary Table 1).

**Table 3:** Criteria of HIQ and questions used in the survey.

**Criteria**	**Definition**	**Question**	**Notes**
Advertisement	Presence of many advertisements	I trust a health webpage more if it has few advertisements	H8
Advertising policy	Clear advertising policy	I trust a health website more if it has a clear advertising policy (a link for advertising policy)	H8, J13
Affiliation	Author affiliation	I trust a health webpage more if it identifies author's affiliation or organization	J1, H1
Authority	Website domain information	I look at the URL of the website and use the domain information (.gov,.com, etc.) to help me determine whether the website is reliable	
Authorship	Author name	I trust a health webpage more if it identifies the author	J1
Complementarity	Disclaimer that information complement doctor	I trust a health webpage more if the website has a disclaimer (usually mentioning they support, not replace, the relationship between patient and physician)	H2
Conciseness	Concise information	I ignore webpages that contain too much information	J2
Copyright	Copyright notice	I trust a health website more if it has a copyright notice	
Currency	Date of information	I trust a health webpage more if it identifies a date	J4, D5
Financial disclosure	Financial disclosure	I trust a health webpage more if the website discloses the owner/ sponsor /source of funds	J3, H7
Focus	Focusing on main topic	I ignore webpages that do not focus on the main topic I am looking for	D3
Grammar	Free of grammatical errors	I trust a health webpage more if its content is free of grammatical errors	
Hyperbole	Existence of easy solutions words	I don't trust websites that offer quick and easy solutions to my health problem with exaggerated words (miracle cures, exaggerated claims, sensational news)	
Instructions	Explain how to take medications.	I prefer webpages that explain how to take the medications	
Multimedia	Existence of videos/pictures	When a search engine returns a list of pages, I select a page from the list based on whether it has video/pictures	
Objectivity	Free from bias or financial interest	I trust a health webpage more if the information it contains is free from bias or financial interest	H5, D6
Payment information	Asks for payment information	I don't trust websites that ask for payment information	
Privacy	Privacy policy	I trust a health website more if it has a clear privacy policy on how my personal information (including those collected automatically by cookies, history or various forms of tracking) is stored and handled	H3
Ranking	Search engine ranking	When a search engine returns a list of pages, I select a page from the list based on their ranking	
Readability	Easy to read	I prefer webpages that are easy to read	
Side effects	Mentions side effects	I prefer webpages that describe any side effects a treatment may cause.	D11
Sources	Existence of sources of information	I trust a health webpage more if it discloses its sources of information	J2, H4, D4
Spelling	Free of spelling errors	I trust a health webpage more if its content is free of spelling errors	
Symptoms	Mention of symptoms	I prefer webpages that explain the symptoms of the disease	
Transparency	Existence of contact information	I trust a health webpage more if the website provides contact information including postal address/telephone (contact us page)	H6
Treatments	Mention of possible treatments	I prefer webpages that suggest the possible treatments to the disease	
Understandability	Easy to understand information	I prefer webpages that are easy to understand (do not use a technical language)	

*Notes indicate when criteria are derived from DISCERN (D), HON (H), or JAMA (J) criteria, referring to numbers in Table 1*.

The project was approved on 26/01/2017 by the Research Ethics Panel of the School of Computer Engineering and Mathematics of the University of Brighton. The questionnaire was published online using Google forms and promoted using social media such as Twitter, Facebook and via email, including students and staff at the University of Brighton and students at the Brighton and Sussex Medical School. We set the Google forms to limit one response per user to avoid duplicate responses. Eligibility criteria for participation were: understanding English language and age over 18. A total of anonymous 329 responses were recorded in the period 1/2/2017–16/6/2017. We considered this a sufficient number as previous studies in the field of IQ and its dimensions are based on surveys with a number of responses ranging from 235 to 355 (10, 11, 15).

Statistical analysis of the responses was performed using SPSS and the specific test is described in the legend of each figure or table. Hierarchical cluster analysis of questionnaire responses (average linkage clustering using the weighted pair group method with arithmetic mean) was performed using GENE-E (Broad Institute, Cambridge, MA) for Windows.

## Results

### Sample characteristics

We received 329 responses, 66% male and 33.7% female. Age groups were: 18–25 years, 26.4%; 26–40, 52.3%; 41–60, 18.8%; over 60, 1.5%. The responses came from 32 different countries: United Kingdom 41.5%, Yemen 20.4%, Saudi Arabia 13.4%, Germany 5.1%, Canada 3.8% and 15.8% various other countries. Of the respondents, 49.5% had, or were studying toward, a postgraduate degree, 40.7% another higher education diploma and 9.8% high school; 26.5% were of a biomedical background (a degree or studying toward a degree in medicine, pharmacology or biomedical sciences). Ten out of 329 participants responded that they do not seek health information online, and these were excluded from the analyses.

### Ranking of IQ criteria

**Figure 1:**
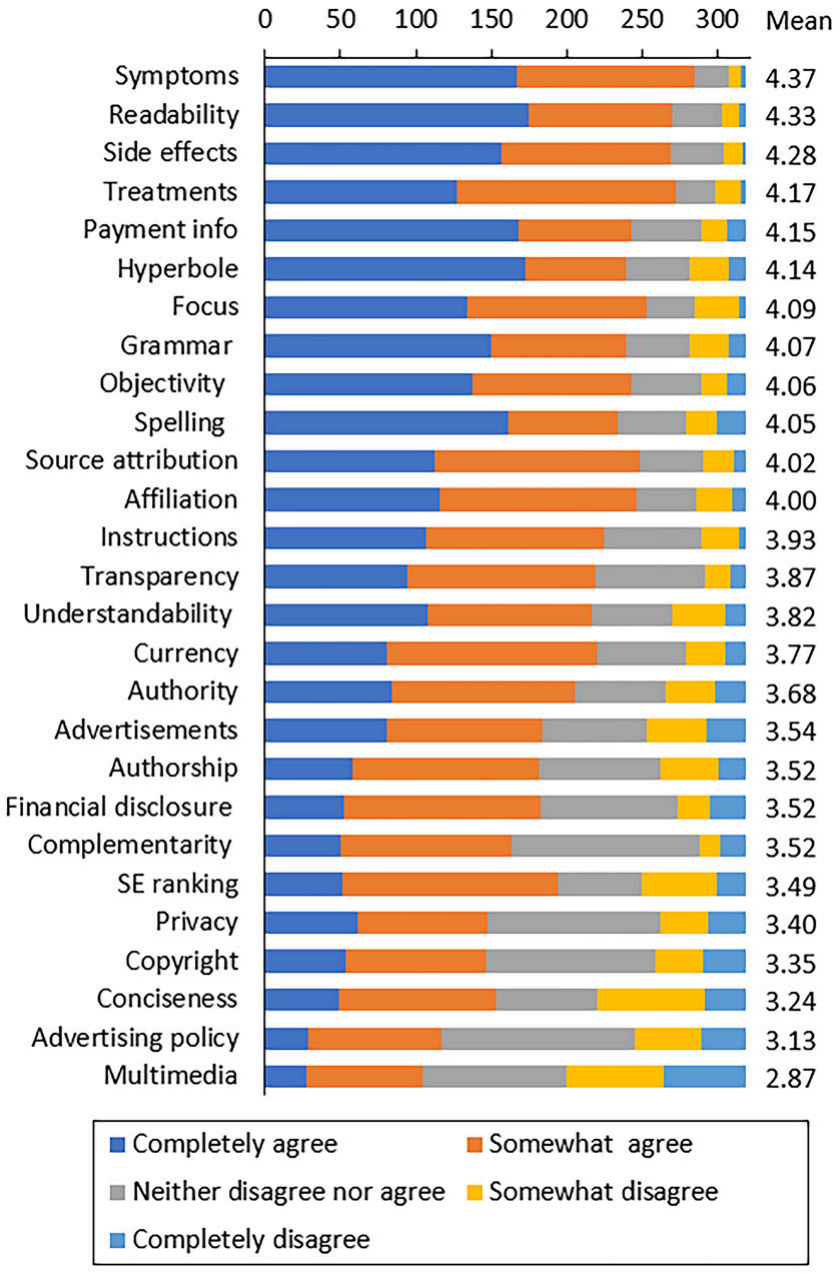
Ranking of HIQ criteria based on questionnaire responses. The horizontal axis indicates the number of responses (total, 319). Criteria are ranked based on the average of the mean Likert scale (right).

Figure 1 show how all respondents ranked each of the IQ criteria described in Table 3. The full results of the questionnaire (raw data, mean, median) are provided as a supplementary file (Supplementary File 1). All responses had a satisfactory inter-rater reliability, with an overall Cronbach's Alpha for all 27 questions of 0.882 (for individual questions, Cronbach's Alpha ranged between 0.874 and 0.883).

The ranking by the average Likert score is shown in Table 4 (first two columns). The median score of all 27 responses listen here was 3.87. It can be seen that a group of criteria that relate to the very specific context of health and disease (symptoms, side effects, treatments and instructions; in bold-italics in Table 4) are ranked high, indicating that users want information that is, above all, relevant and helpful.

**Table 4:** Ranking of criteria by perceived importance.

**All**	**Mean**	**Biomedical**	**Mean**	**Non-Biomedical**	**Mean**	**English**	**Mean**	**Non-English**	**Mean**	**Male**	**Mean**	**Female**	**Mean**
***Symptoms***	4.37	***Symptoms***	4.45	***Symptoms***	4.34	Hyperbole	4.49	Readability	4.33	***Symptoms***	4.33	*Spelling*	4.58
Readability	4.33	*Spelling*	4.43	Readability	4.32	***Symptoms***	4.44	***Symptoms***	4.31	Readability	4.29	*Grammar*	4.58
***Side effects***	4.28	***Side effects***	4.40	***Side effects***	4.24	***Payment info***	4.42	***Side effects***	4.28	***Side effects***	4.27	***Symptoms***	4.45
***Treatments***	4.17	*Grammar*	4.39	***Treatments***	4.16	*Spelling*	4.40	*Focus*	4.21	***Treatments***	4.14	*Hyperbole*	4.44
**Payment info**	4.15	Readability	4.36	**Payment info**	4.13	*Grammar*	4.39	***Treatments***	4.09	Focus	4.12	***Objectivity***	4.43
Hyperbole	4.14	Hyperbole	4.35	Focus	4.08	***Objectivity***	4.34	*Understandability*	4.03	Affiliation	4.03	**Payment info**	4.41
Focus	4.09	*Sources*	4.23	Hyperbole	4.07	Readability	4.33	***Instructions***	4.03	**Payment info**	4.02	Readability	4.41
Grammar	4.07	**Objectivity**	4.23	**Objectivity**	4.00	***Side effects***	4.29	Affiliation	3.96	***Instructions***	4.02	***Side effects***	4.31
**Objectivity**	4.06	**Payment info**	4.21	Grammar	3.96	***Treatments***	4.26	Transparency	3.93	*Understandability*	4.01	***Treatments***	4.23
Spelling	4.05	***Treatments***	4.21	Affiliation	3.95	***Sources***	4.24	**Payment info**	3.92	Hyperbole	4.00	*Sources*	4.20
Sources	4.02	Affiliation	4.16	Sources	3.95	Affiliation	4.05	Hyperbole	3.84	Sources	3.91	Focus	4.05
Affiliation	4.00	Focus	4.11	*Understandability*	3.94	Focus	3.95	Sources	3.82	Transparency	3.89	Affiliation	3.96
***Instructions***	3.93	***Instructions***	3.95	Spelling	3.93	***Instructions***	3.82	**Objectivity**	3.81	**Objectivity**	3.86	Transparency	3.80
Transparency	3.87	Currency	3.93	***Instructions***	3.92	*Currency*	3.81	Grammar	3.78	Grammar	3.80	***Instructions***	3.78
Understandability	3.82	Transparency	3.84	Transparency	3.87	Transparency	3.79	Currency	3.75	*Currency*	3.79	Currency	3.77
Currency	3.77	**Advertisement**	3.75	*Authority*	3.79	Authority	3.70	Spelling	3.75	Spelling	3.78	**Complementarity**	3.69
Authority	3.68	**Financial disclosure**	3.69	***Currency***	3.72	**Advertisements**	3.66	Authority	3.65	Authority	3.73	**Financial disclosure**	3.68
**Advertisement**	3.54	Authorship	3.59	**Complementarity**	3.50	**Financial disclosure**	3.60	***Copyright***	3.61	SE ranking	3.59	**Advertisements**	3.68
Authorship	3.52	**Complementarity**	3.58	Authorship	3.49	Understandability	3.58	SE ranking	3.59	***Copyright***	3.55	Authority	3.59
**Financial disclosure**	3.52	SE ranking	3.49	SE ranking	3.49	**Complementarity**	3.57	**Privacy**	3.52	Authorship	3.50	Authorship	3.55
**Complementarity**	3.52	Understandability	3.46	**Advertisements**	3.47	Authorship	3.56	Authorship	3.48	**Privacy**	3.50	Understandability	3.48
SE ranking	3.49	**Privacy**	3.45	**Financial disclosure**	3.46	SE ranking	3.38	**Complementarity**	3.47	**Advertisements**	3.47	SE ranking	3.32
**Privacy**	3.40	Authority	3.35	**Copyright**	3.39	**Privacy**	3.26	**Financial disclosure**	3.44	**Financial disclosure**	3.44	**Privacy**	3.23
**Copyright**	3.35	**Copyright**	3.21	**Privacy**	3.38	**Advertising policy**	3.09	**Advertisements**	3.44	**Complementarity**	3.43	**Advertising policy**	3.09
Conciseness	3.24	**Advertising policy**	3.13	**Conciseness**	3.28	**Copyright**	3.05	Conciseness	3.41	*Conciseness*	3.35	Conciseness	3.05
**Advertising policy**	3.13	Conciseness	3.10	**Advertising policy**	3.13	Conciseness	3.04	Multimedia	3.26	**Advertising policy**	3.15	**Copyright**	3.00
Multimedia	2.87	Multimedia	2.73	Multimedia	2.92	Multimedia	2.42	**Advertising policy**	3.16	Multimedia	3.08	Multimedia	2.48

*The first column lists the criteria ranked in order of importance. Values are the mean of the Likert scale. Green (italics bold in print) denotes criteria belonging to “completeness”; yellow (shaded-bold in print) those in “ethics.” Criteria in red (underlined in print) fonts are significantly higher when comparing two groups as described in the text (P < 0.05 by ANOVA test)*.

On the other hand, criteria related to the four JAMA criteria (authorship, currency, sources, financial disclosure) are not considered particularly important and, with the only exception of “sources,” are all ranked below the median value.

Of the 8 criteria related to the HONcode principles, only one was slightly above the median (affiliation; termed “authority” in the HON principles) while all the others (complementarity, privacy, attribution/sources, transparency, financial disclosure, advertising policy) were not deemed highly important (one criterion, “justifiability,” was not assessed in the questionnaire). With the exception of “sources,” a criterion that belongs to those in the JAMA criteria, all the criteria above could be broadly related to “ethics” and are highlighted in bold in Table 4. Authority, which we define as the affiliation of the website—whether governmental, from an international health organization, for instance (while we define affiliation as that of the author) also ranked low.

### Identification of main dimensions of HIQ

We attempted to group the various criteria in IQ dimensions. To do so we have used a mixed approach. In part we relied on an ontological/theoretical approach and the existing classification described in Table 2.Then, with an empirical approach, we assessed whether some of these criteria followed a similar pattern in the responses to the questionnaire. For this purpose, we analyzed all individual responses using hierarchical cluster analysis.

**Figure 2:**
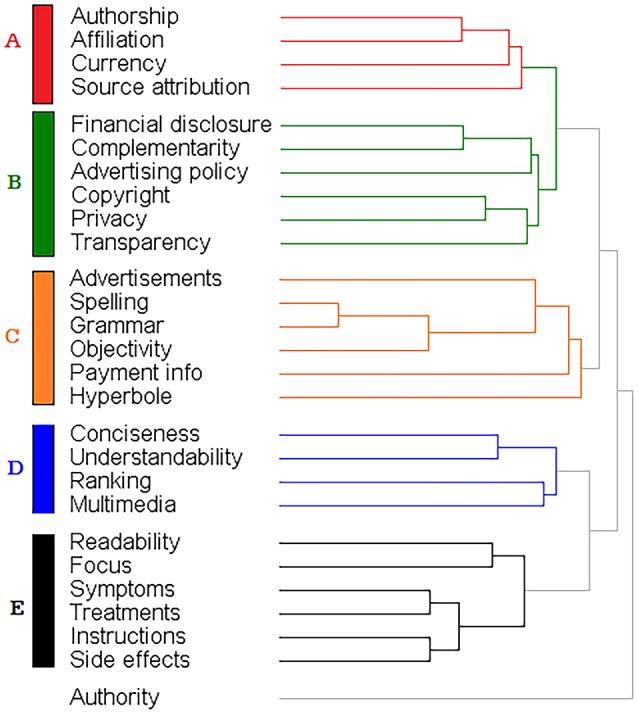
Clusters of HIQ criteria. Hierarchical cluster analysis of the Likert scale score for different criteria among 319 participants.

As shown in Figure 2, we identified five main clusters. Cluster A includes three of the JAMA criteria (authorship, currency and sources) and affiliation. Cluster B includes financial disclosure, complementarity, advertising policy, copyright, privacy and transparency, all criteria that somewhat relate to ethical aspects of IQ. Cluster C includes basic features of webpages (number of advertisements, spelling, grammar and objectivity) as well as hyperbole and payment info. Cluster D includes IQ criteria (conciseness, ranking, and multimedia) that specifically relate to online information in addition to understandability.

Cluster E includes criteria that relate to the practical usefulness for an information seeker in the specific context of health and disease (focus, symptoms, treatments, side effects of drugs, and information on their usage). This cluster also includes readability and although at first one may think that this is a feature of the text (like spelling or grammar) it has probably a more practical value.

We now propose an organization of criteria of HIQ into dimensions, as outlined in Table 5. A first dimension relates to trustworthiness but could be better defined as “accountability” and includes information that defines basic criteria such as not being anonymous. This dimension includes four of the components of the JAMA score that are present in cluster A. We also included in this dimension “authority” that did not belong to any cluster. In fact, our questionnaire defined authority as features of a website (such as the domain, whether.com, edu or.org) and this is very similar to “affiliation,” defined as the affiliation of the individual author. We also included in this dimension “transparency” because, although in cluster B, it was defined as the presence of contact information for the author or website. The criteria of accountability are all intrinsic dimensions of HIQ and would apply equally well to information online and in print and would also apply to non-health related information.

**Table 5:** Proposed criteria and dimensions of HIQ.

**Dimension**	**Criteria**	**Dimension**	**Criteria**
Accountability	Authorship Affiliation Currency Source attribution Transparency (contact info) Authority	Accuracy (textual)	Spelling Grammar Hyperbole Readability
		Representational	Conciseness Understandability Ranking in SERP Multimedia (presence)
Ethics	Financial Disclosure Complementarity Advertising policy Copyright Privacy Advertisements (presence of) Objectivity (free from COI^*^) Payment information request		
		Completeness/ Purposeness	Symptoms Treatments Instructions Side effects Focus

* *COI, conflict of interest*.

A second dimension, *ethics*, defines ethical aspects of trustworthiness and includes all the criteria in cluster B except transparency (see above). We also included here “objectivity,” “advertisement,” and “payment information” although they clustered elsewhere, as this would fit with the description of this dimension. These are criteria of HIQ that could also be applied to non-health IQ with the exception of complementarity (the presence of a statement to say information supports, does not replace, the relationship between patient and physician). Financial disclosure might be important in other types of information, but the issue of funding and conflict of interest is regarded as particularly important in health.

A third dimension defines *textual accuracy* and includes spelling, grammar, readability and use of hyperbole or exaggeration. To define this dimension, we started from cluster C. However, because “hyperbole” can be considered a characteristic of the text, we decided to subsume it under “accuracy.” This dimension could apply equally to non-health, and in print, information, with the possible exception of hyperbole or exaggeration, that is more common in the news about scientific advancements.

A fourth dimension, defined as “*representational*” dimension comprises criteria (understandability, conciseness, search engine ranking and presence of multimedia) that is probably more important in online information (that one wants to access quickly and concisely, so it can be read on a small screen) but would apply to non-health subjects. These criteria are present exactly in cluster D.

A last dimension defines the much sought-after elements of information that characterize its scientific *completeness*: presence of information specific to the medical condition or its treatment, as well as focus. In fact, all these criteria relate to focus. As such, even if these specific criteria relate to health, it would be easy to identify homologous criteria in other fields. Also, this dimension could also apply to printed information although focus is probably more important when information is accessed online, often on a small mobile device.

### Subgroup analysis by educational subject, gender and language

We first analyzed differences in the ranking given by participants based on whether they studied, or had a degree, in a biomedical field or not. Then we looked at native language (English vs. non-English) and gender.

The results are shown in Table 4 that reports, in columns 3rd to 14th, the ranking (as mean score) for all subgroups. When comparing biomedical students/graduates with non-biomedical ones, it was clear that biomedical education was associated with giving higher importance to text accuracy (spelling, grammar, sources). Higher importance to text accuracy (spelling, grammar, hyperbole) was also evident for English speakers, compared to non-English. There were also significant gender differences with textual accuracy being ranked higher in females, while males ranked higher “instructions” and “understandability.”

### Importance of the scientific correctness of the information provided

We have noted earlier that information about disease diagnosis and treatment is ranked highest in the whole sample (in the top quartile). However, the fact that a web page describes a treatment for a disease does not mean that website is scientifically correct. One could come up with a web page that meets all the criteria in the “completeness” dimension but misinforms the reader.

We recently proposed to use the information about the treatment suggested or promoted as a proxy for the scientific soundness of a web page (16). Therefore, we asked participants whether they prefer websites that provide EBM information, complementary or alternative medicine (CAM), or don't care. The results shown in Table 6 indicate that only 6% preferred websites on CAM, 35% preferred EBM and 37% did not assign this a particular importance. However, the preference for EBM was higher with biomedical education, English speakers and females, and in these groups, there was a lower percentage of participants who did not know whether they prefer EBM or CAM. The association with biomedical education, language and gender was statistically significant (*P* = 0.02, *P* < 0.001, *P* = 0.029, respectively, by the Pearson Chi-Square test). There was no significant association with EBM preference and education level (*P* = 0.866, data not shown).

**Table 6:** Preference for EBM- or CAM-based information.

	**EBM**	**CAM**	**Don't mind both**	**Don't know**	**Total**
All	35% (110)	6% (20)	37% (118)	22% (70)	(318)
**SUBJECT**					
Med	41% (33)	5% (4)	44% (35)	10% (8)	(80)
Non-med	32% (77)	7% (16)	35% (84)	26% (62)	(239)
**LANGUAGE**					
English	40% (59)	3% (4)	44% (66)	13% (20)	(149)
Non-English	30% (51)	9% (169)	31% (53)	29% (50)	(170)
**GENDER**					
Male	32% (67)	8% (16)	34% (70)	26% (54)	(207)
Female	39% (43)	4% (4)	43% (48)	14% (16)	(111)

*Data indicate the percentage of responders, number in parentheses are the absolute number*.

## Discussion

We propose dimensions and criteria of HIQ based on the importance assigned to them by internet users. We used an empirical approach, like what was done 20 years ago by Wang and Strong (10) and Lee (11) for IQ in the context of industries and organizations, with two major differences: the focus on the health-related content of the information provided by websites and that on trustworthiness, and that on online information. The results were not only used to rank the different criteria in order of perceived importance but also, using cluster analysis, to help with classifying them into dimensions.

Although the terminology is always ambiguous, we suggest that criteria of HIQ could be subsumed to dimensions as described in Table 5, bearing in mind that there may be area of overlaps. For instance, we assigned the criterion “hyperbole,” that in the context of HIQ means presenting a potential treatment as a “miracle drug,” to the dimension of textual accuracy but on theoretical grounds it could also fit the ethical dimension of trustworthiness.

Of the criteria in the dimension “accountability,” which includes the four JAMA criteria (authorship, currency, sources financial disclosure), “sources” is the one that ranks highest, but sill only 11th. Authorship (19th) ranked lower than authority (17th) and affiliation (12th), indicating that the link to an institution or a medical degree, or the type of website (for instance whether a government website or a commercial one) are considered more important than the indication of the name of the author. The generally low importance given to the JAMA criteria was also observed in a survey by Eysenbach and Kohler (17) as they reported that “Contrary to the statements made in the focus groups, in practice we observed that none of the participants actively searched for information on who stood behind the sites or how the information had been compiled” (17).

The ethics dimension of trustworthiness includes aspects that are particularly important in medicine (conflict of interest, data privacy, financial disclosure). Of note, one criterion, “complementarity” [whether Information should support, not replace, the doctor-patient relationship) is one of the HONcode principles (3)] and specific for health.

The contextual information that we define as “textual accuracy” are also ranked high, and these include spelling and grammar but also include the health-specific criterion “hyperbole,” that is very common in health news stories and web pages when authors portray a treatment with an overly positive tone or “spin” (18).

The “completeness” dimension defines contextual information [that is necessary for the information to fulfill its task (19)]. It includes both basic IQ criteria as well as some that are specific to health, and we could define it as “scientific completeness,” the information that users look for and rank high in our questionnaire. This is in agreement with a recent study performed in the US showing that completeness of the information, that the authors defined as “the proportion of priori-defined elements covered by the website; breath of information” also ranked higher in a study where participants were asked to rank health websites for some IQ criteria (12). The importance given by participants to criteria related to “completeness/purposeness,” as indicated by the high ranking of information on symptoms, side effects and treatments in Table 4 reflects the main use of the internet when searching health information. In fact, a survey of 622 patients in the MetroNet practices in the Detroit area reported that of the topics most often searched online, specific disease conditions and treatments come on top (20). To “find out about treatments” was also the top purpose of health-related internet use in a survey of patients of a general practice surgery in semi-rural England (21).

Of the representational criteria, understandability ranked rather high. On the other hand, representational criteria specific for webpages (ranking by the search engine, presence of multimedia, conciseness) are deemed as the least important.

Another aspect highlighted by the present study is that the ranking of criteria of HIQ is not a one-size-fits-all but differs depending on education, gender and linguistic background. This is not a novel concept, and already Wang and Strong suggested that the classification of IQ criteria in dimensions is different for academic and practitioners, in a way, an extension of the concept of data “fit-for-use” (10). Floridi also noted that IQ should also consider purposeness, and that the value of IQ criteria may be different in different users (22).

In this sense, the difference in the ranking of HIQ criteria among subjects with a biomedical degree or biomedical students (pharmacy, biomedical science, medicine) and those in other education areas could be extrapolated to the difference between health professionals and lay persons. Those with a biomedical background give more importance to criteria such as correct spelling and grammar than those with non-biomedical background. Not surprisingly, “sources” are ranked higher in a biomedical background, as identifying and citing references is key to this field. On the other hand, in a non-biomedical background, “understandability” is ranked higher. Interestingly, we have not found any significant difference in the ranking of the “ethical” criteria by subjects with a biomedical background.

Native English speakers also assign more importance to textual accuracy (spelling, grammar,), as well as to the ethical criterion of “objectivity.” Attention to “hyperbole” is also ranked higher by this group and we discussed above how this criterion has also an “ethical” value. A very similar pattern was observed in females, when compared with males, with the added higher importance assigned to “payment information,” suggesting a stronger ethical focus in females.

The differences in ranking identified in the subgroup analysis hint at a limitation of any classification into dimensions based on a questionnaire, as the results will vary with the population investigated, and any subsequent analysis (including the cluster analysis used here) will vary accordingly. This suggests that when IQ is defined, the target user should be well defined.

The other aspect of this study was on which criteria are regarded as important and which are not. The fact that the ranking by the search engine is not seen as an indicator of trustworthiness of a website is very interesting, but this does not mean that the user is likely to go through several search engine result pages rather than limiting to the first 10–20. The significance of this response should be assessed experimentally, for instance using eye-tracking software to validate the importance of the different criteria.

The low ranking on “ethical” trustworthiness criteria is worrying as it might indicate that users are somewhat vulnerable to information that has a conflict of interest, such as that from commercial sources promoting potentially ineffective treatments or to other types of health misinformation. This is probably something that educators, particularly those in the bio-medical field, should consider improving, and males seem to be more “at risk” as they value “ethical” criteria lower than females. This difference is supported by a recent study reporting that males are more likely to disseminate fake health information than females (23). It should be noted that this is at variance with results from the MetroNet study cited above, where patients ranked “Endorsement by a government agency or professional organization” and “reliable source/author” as the most important factors influencing their trust (“perceived accuracy” of healthcare websites (20). Likewise, “reputable/trustworthy organization” was the most important factor in trusting health information in a 2002–2003 survey of 55 participants to UK health support groups, although this study was not restricted to online information but included also information provided by healthcare professionals, brochures, books, TV/radio, and others (24). It is difficult to say whether these differences are due to the different time periods when those studies were carried out or whether this is due to the difference in the population, and patients exposed to medical research and support groups may have a higher health literacy than our sample.

We suggest that our proposed dimensions of HIQ may be an attempt to build a more comprehensive theoretical framework than the one that can be derived from the existing studies. For instance, the recent paper by Tao et al. (12) proposing a definition of HIQ dimensions does not take into account some of the criteria that we derived from the HONcode and DISCERN tools, particularly those related to what we call “ethical criteria” (12).

In conclusion, this study describes a possible organization of HIQ criteria into dimensions that identifies dimensions not previously recognized as such in IQ, such as the ethical dimension which was identified through this ranking approach. Contrary to our expectations, given this is a hot topic in the news, we observed that ethical criteria, while regarded highly in the academic and medical environment, are not considered highly by the public.

Clearly, the main limitation of this study, which could affect its external validity, is that the focus on university-level participants mainly may lead to an underestimation of the importance of criteria aimed at the average user. It would be important to extend this study to a more general sample of the public, and particularly patients and carers, to see whether there is a different perception of HIQ and if this goes in the same direction of the results in the comparison between non-biomedical and biomedical educational background reported here. Another important point to consider when extrapolating the conclusions of this study is that our survey asked generically what users would look for to trust a website when searching for a health topic. It is possible that the factors that account for trust in a webpage with health-related information is different depending on the topic searched, and this may be particularly important for highly controversial topics, such as abortion, vaccines or genetic modifications.

## Author contributions

All authors designed research, analyzed the data, wrote the paper. MA-J designed research, performed research, analyzed the data, wrote the paper.

### Conflict of interest statement

The authors declare that the research was conducted in the absence of any commercial or financial relationships that could be construed as a potential conflict of interest.
