# Supplementary material for: What Is Health Information Quality? Ethical Dimension and Perception by Users
Source: Front Med (Lausanne). 2018 Sep 20;5:260. doi: 10.3389/fmed.2018.00260 (PMC6158347; doi:10.3389/fmed.2018.00260)
Supplement: Supplementary file 1 [file Table_1.DOCX]

# Supplementary Table 1: Survey Questions

| **Question** | **Answers** | | | | | |
| --- | --- | --- | --- | --- | --- | --- |
| **Your use of Internet to search information:** |  | | | | |  |
| 1) How many hours (per week) do you spend searching for information on the web? | Less than 2 hours | 2-6 hours | 7-12 hours | More than 12 hours |  |  |
| 2) How many years since you became familiar with Internet? | Less than three years | 3-5 years | 5-10 years | More than 10 years |  |  |
| 3) What device do you use to browse Internet (you can select more than one)? | Personal Computer | Tablet | Smart phone | Smart TV | Other: please specify |  |
| 4) Which search engine do you mainly use? | Google | Bing | Yahoo! | Other: please specify |  |  |
| 5) In the last year, how often have you tried to seek healthcare information online (for you or for someone else)? | 1-2 times | 3-5 times | More than 6 times | I have never sought health information online |  |  |
| 6) Please specify the purpose of seeking health information online. Is it to : (you can select more than one answer) | Look for medication for specific disease | Look for symptoms and the possible diagnosis | Read more about disease diagnosed by your doctor | Read about medications prescribed by your doctor | I never seek HI | Other: please specify |
| 7) How do you start looking for health information online (think about the last time you did this)? Do you start: | At a general search engine such as Google, Bing or Yahoo | At a site that specializes in health information, like NHS choices or WebMD (if you use search engine to reach a specific site select this option) | At a more general site like Wikipedia, that contains information on all kinds of topics (if you use search engine to reach a specific site select this option) | At a social network site like Facebook | Other: please specify |  |
| 8) Do you believe that pages returned by your search engine are the best matches for your search term. | Completely  disagree | Somewhat disagree | Neither disagree nor agree | Somewhat agree | Completely  agree |  |
| 9) If you make a query in a search engine, how many results do you usually view? | Just one link | 2-3 links | 4-5 links | More than 5 links |  |  |
| **Your trust in online health information:** |  | | | | |  |
| 1) I trust a health webpage more if it identifies the author. | Completely  disagree | Somewhat disagree | Neither disagree nor agree | Somewhat agree | Completely  agree |  |
| 2) I trust a health webpage more if it identifies author’s affiliation or organization (PhD, M.D., University. Hospital, etc.) | Completely  disagree | Somewhat disagree | Neither disagree nor agree | Somewhat agree | Completely  agree |  |
| 3) I trust a health webpage more if it identifies a (written/updated/reviewed) date | Completely  disagree | Somewhat disagree | Neither disagree nor agree | Somewhat agree | Completely  agree |  |
| 4) I trust a health webpage more if it discloses its sources of information. | Completely  disagree | Somewhat disagree | Neither disagree nor agree | Somewhat agree | Completely  agree |  |
| 5) I trust a health website more if it has a copyright notice | Completely  disagree | Somewhat disagree | Neither disagree nor agree | Somewhat agree | Completely  agree |  |
| 6) I trust a health website more if it has a clear privacy policy on how my personal information (including those collected automatically by cookies, history or various forms of tracking) is stored and handled (for example via a privacy link). | Completely  disagree | Somewhat disagree | Neither disagree nor agree | Somewhat agree | Completely  agree |  |
| 7) I trust a health webpage more if the website discloses the owner/sponsor/source of funds. | Completely  disagree | Somewhat disagree | Neither disagree nor agree | Somewhat agree | Completely  agree |  |
| 8) I trust a health webpage more if the website has a disclaimer (usually mentioning they support, not replace, the relationship that exists between a patient and his/her existing physician) | Completely  disagree | Somewhat disagree | Neither disagree nor agree | Somewhat agree | Completely  agree |  |
| 9) I trust a health website more if it has a clear advertising policy (usually there is a link for advertising policy). | Completely  disagree | Somewhat disagree | Neither disagree nor agree | Somewhat agree | Completely  agree |  |
| 10) I trust a health webpage more if it has few advertisements. | Completely  disagree | Somewhat disagree | Neither disagree nor agree | Somewhat agree | Completely  agree |  |
| 11) I trust a health webpage more if its contents are free of spelling errors. | Completely  disagree | Somewhat disagree | Neither disagree nor agree | Somewhat agree | Completely  agree |  |
| 12) I trust a health webpage more if its contents are free of grammatical errors. | Completely  disagree | Somewhat disagree | Neither disagree nor agree | Somewhat agree | Completely  agree |  |
| 13) I trust a health webpage more if the information it contains is not subjective (free from bias or financial interest) | Completely  disagree | Somewhat disagree | Neither disagree nor agree | Somewhat agree | Completely  agree |  |
| 14) I trust a health webpage more if the website provides contact information including postal address/telephone (contact us page). | Completely  disagree | Somewhat disagree | Neither disagree nor agree | Somewhat agree | Completely  agree |  |
| 15) I prefer webpages that are easy to read | Completely  disagree | Somewhat disagree | Neither disagree nor agree | Somewhat agree | Completely  agree |  |
| 16) I ignore webpages that do not focus on the main topic I am looking for | Completely  disagree | Somewhat disagree | Neither disagree nor agree | Somewhat agree | Completely  agree |  |
| 17) I ignore webpages that contain too much information | Completely  disagree | Somewhat disagree | Neither disagree nor agree | Somewhat agree | Completely  agree |  |
| 18) I prefer webpages that are easy to understand (do not use a technical language) | Completely  disagree | Somewhat disagree | Neither disagree nor agree | Somewhat agree | Completely  agree |  |
| 19) I look at the URL of the website and use the domain information (.gov,.edu, .org, .com, etc.) to help me determine whether or not the website is reliable. | Completely  disagree | Somewhat disagree | Neither disagree nor agree | Somewhat agree | Completely  agree |  |
| 20) When a search engine returns a list of pages, I select a page from the list based on their rank provided by the search engine | Completely  disagree | Somewhat disagree | Neither disagree nor agree | Somewhat agree | Completely  agree |  |
| 21) When a search engine returns a list of pages, I select a page from the list based on whether or not it has video/pictures | Completely  disagree | Somewhat disagree | Neither disagree nor agree | Somewhat agree | Completely  agree |  |
| 22) I don’t trust websites that ask for payment information. | Completely  disagree | Somewhat disagree | Neither disagree nor agree | Somewhat agree | Completely  agree |  |
| 23) I prefer webpages that explain the symptoms of the disease I am looking at. | Completely  disagree | Somewhat disagree | Neither disagree nor agree | Somewhat agree | Completely  agree |  |
| 24) I prefer webpages that suggest the possible treatments to the disease | Completely  disagree | Somewhat disagree | Neither disagree nor agree | Somewhat agree | Completely  agree |  |
| 25) I prefer webpages that explain how to take the medications. | Completely  disagree | Somewhat disagree | Neither disagree nor agree | Somewhat agree | Completely  agree |  |
| 26) I prefer webpages that describe any side effects a treatment may cause. | Completely  disagree | Somewhat disagree | Neither disagree nor agree | Somewhat agree | Completely  agree |  |
| 27) I don’t trust websites that offer quick and easy solutions to my health problem with exaggerated words (for example miracle cures, exaggerated claims, sensational news) | Completely  disagree | Somewhat disagree | Neither disagree nor agree | Somewhat agree | Completely  agree |  |
| 28) I prefer a health webpage if it suggests: | Medicine of which the effectiveness was demonstrated scientifically in clinical trials. | Only alternative medicine (any of a range of medical therapies that are not regarded by medical professionals) | I don’t mind if it has both 1 & 2 | I don’t know |  |  |
| Please provide any further comments (please suggest any features you think we didn’t consider) |  | | | | |  |
| **About you:** |  | | | | |  |
| What is your gender? | Male | Female | Prefer not to say |  |  |  |
| What is your age group? | 18-25 | 26-40 | 41-60 | >60 | Prefer not to say |  |
| What is your country if residence? |  | | | | |  |
| What is your education level? | High school | - Graduate/higher diploma | Postgraduate |  |  |  |
| What is your subject area? (if applicable) | Engineering/Math related | Medical related | Business related | Arts related | *Others please specify* |  |
